# New drugs in the treatment of dual psychosis: use of cariprazine in schizophrenia, other psychotic disorders and use of cocaine. A case series in a specific outpatient psychiatric clinic for substance use disorders.

**DOI:** 10.1192/j.eurpsy.2024.613

**Published:** 2024-08-27

**Authors:** G. Montero-Hernandez, I. Alberdi-Páramo, M. Pérez-Lombardo, J. Rodríguez-Quijano, J. Pemán-Rodríguez, J. E. Ibáñez-Vizoso

**Affiliations:** ^1^Red Salud Mental Bizkaia, Osakidetza, Bilbao; ^2^Instituto de Psiquiatría y Salud Mental, Hospital Clínico San Carlos, Madrid; ^3^Servicio de Psiquiatría, Hospital Universitario Nuestra Señora de Candelaria, Tenerife; ^4^Servicio de Psiquiatría, Complejo Hospitalario Universitario de Vigo, Vigo, Spain

## Abstract

**Introduction:**

New drugs in the treatment of dual psychosis: use of cariprazine in schizophrenia, other psychotic disorders and use of cocaine. A case series in a specific outpatient psychiatric clinic for substance use disorders.

**Objectives:**

The main objective of this case series is to observe and describe the tolerability and clinical response to different doses of cariprazine in a series of patients with dual psychosis, especifically cocaine users; with a special attention upon psychotic symptoms, disruptive behaviour, affective symptoms and cocaine use pattern.

**Methods:**

This series consists of an observation of a total of 20 patients treated on an outpatient basis. All of them had a either a diagnosis of Schizophrenia or Other Non Specified Psychotic Disorder meeting the DSM-5 criteria, as well as a Cocaine Use Related Disorder meeting the DSM-5 criteria. All of them received treatment with cariprazine in different doses from 1,5mg to 6mg per day, as a solo treatment or as an adjuvant to another previous antipsychotic treatment when antipsychotic augmentation was justified. We observed patients that had started cariprazine in the past three months and that had active drug use or had had one in the past three months.

We monitored the tolerance to the treatment, the clinical response in terms of positive and negative symptoms of schizophrenia, affective symptoms, disruptive behavior, and the response in terms of substance use; for a period of six months of follow-up, with psychiatric consultation at least every month and nurse consultation every two weeks in our clinic.

**Results:**

95% of the patients did not present any side effect related to cariprazine. In one patient (5%) the treatment had to be stopped due to akathisia that did not disappear after two weeks and symptomatic treatment with benzodiacepines. 60% of patients either stopped using (50%) or reduced their use frequency (50%). 70% of the patients presented an improvement in positive symptoms and behavior. Also, one third of them presented a slight improvement in negative symptoms. 20% of patients referred a significant improve in depressive symptoms.

**Conclusions:**

The main conclusion of this case series is that cariprazine at any dosis between 3mg and 6mg per day has a positive outcome, both in the psychotic domain and the substance use disorder. We hope this case series will help our colleagues treat their patients suffering from these pathologies in an optimal way. This could also set a basis to encourage a proper clinical trial to assess if new antipsychotics such as cariprazine could be a new standard for the treatment of Dual Disorders.

**Disclosure of Interest:**

None Declared

